# Antimicrobial Peptide Reverses ABCB1-Mediated Chemotherapeutic Drug Resistance

**DOI:** 10.3389/fphar.2020.01208

**Published:** 2020-08-07

**Authors:** Xiaofang Luo, Qiu-Xu Teng, Jin-Yun Dong, Dong-Hua Yang, Meifeng Wang, Wubliker Dessie, Jiang-Jiang Qin, Zi-Ning Lei, Jing-Quan Wang, Zuodong Qin, Zhe-Sheng Chen

**Affiliations:** ^1^ Research Center of Biochemical Engineering Technology, College of Chemistry and Bioengineering, Hunan University of Science and Engineering, Yongzhou, China; ^2^ Department of Pharmaceutical Sciences, College of Pharmacy and Health Sciences, St. John’s University, Queens, NY, United States; ^3^ Institute of Cancer and Basic Medicine, Chinese Academy of Sciences, Cancer Hospital of the University of Chinese Academy of Sciences, Zhejiang Cancer Hospital, Hangzhou, China

**Keywords:** multidrug resistance, ABC transporter, antimicrobial peptide HX-12C, reversal agents, combination therapy

## Abstract

Multidrug resistance (MDR) of tumor cells to chemotherapeutic agents is the main reason for the failure of cancer chemotherapy. Overexpression of ABCB1 transporter that actively pumps various drugs out of the cells has been considered a major contributing factor for MDR. Over the past decade, many antimicrobial peptides with antitumor activity have been identified or synthesized, and some antitumor peptides have entered the clinical practice. In this study, we report that peptide HX-12C has the effect of reversing ABCB1-mediated chemotherapy resistance. In ABCB1-overexpressing cells, nontoxic dose of peptide HX-12C inhibited drug resistance and increased the effective intracellular concentration of paclitaxel and other ABCB1 substrate drugs. The mechanism study showed that peptide HX-12C stimulated ABCB1 ATPase activity without changing the expression level and localization patterns of ABCB1. Molecular docking predicted the binding modes between peptide HX-12C and ABCB1. Overall, we found that peptide HX-12C reverses ABCB1-mediated MDR through interacting with ABCB1 and blocking its function without affecting the transporter’s expression and cellular localization. Our findings suggest that this antimicrobial peptide may be used as a novel prospective cancer therapeutic strategy in combination with conventional anticancer agents.

## Introduction

Cancer remains the main cause of human death (Torre et al., 2015; Siegel et al., 2020). Among all kinds of cancer treatments, chemotherapy is still the mainstay therapy for most cancers in clinic (Dong et al., 2018b). Although new anticancer drugs and chemotherapy protocols are being constantly found or optimized, the therapeutic efficacy has not significantly improved. This is mainly because most patients will develop resistance to conventional and targeted chemotherapy during the course of their treatment. Therefore, drug resistance has been considered a major obstacle to the successful treatment of cancers (Holohan et al., 2013; Tsukamoto et al., 2019). Cancer cells can obtain broad-spectrum resistance to anticancer drugs with different structures and mechanisms. This phenomenon is generally known as multidrug resistance (MDR) (Horsey et al., 2016). The molecular mechanisms of MDR are complex, including attenuated intracellular drug concentration by activating the drug efflux pump, as well as upregulation of DNA repair and drug metabolism-related enzymes (Conde et al., 2013; Dong et al., 2018a; Waghray and Zhang, 2018). Among them, transporter-mediated drug resistance caused by overexpression of triphosphate (ATP) binding cassette (ABC) transporters is considered to be a universal mechanism of tumor MDR (Tao et al., 2009).

The ABC superfamily is one of the largest families of transport proteins with 49 different members classified into seven subfamilies from A to G based on their sequence similarities and structure organization (Kumar et al., 2012). Among them, ABCB1 (P-gp/MDR1), ABCCs (MRPs) and ABCG2 (BCRP/MXR/ABCP) are the main members of the ABC transporters related to MDR in tumor cells (Arrigoni et al., 2016; Obreque-Balboa et al., 2016). These transport proteins have the ability to transport a wide range of substrates, such as ions, lipids, drugs, cholesterol, toxins, peptides, and bile salts (Gutmann et al., 2010; Balabanov et al., 2011; Dong et al., 2020). At present, ABCB1 has been the most studied among various transporters because it has been demonstrated as a primary mechanism of resistance in many cancer cells (Teodori et al., 2007; Chen et al., 2015). Therefore, clinical applications of combining an ABCB1 inhibitor and an anticancer drug have been considered to be a promising strategy to circumvent ABCB1-mediated efflux for a long period (Thomas and Helen, 2003; Kozovska et al., 2014). The surface density of many membrane proteins including ABCB1 is regulated by ubiquitination catalyzed by ubiquitin E3 ligases (Akkaya et al., 2015; Wu et al., 2018). Therefore, targeting ubiquitin pathway is a new strategy for against cancers (Soucy et al., 2009; Brahemi et al., 2010; Brownell et al., 2010). However, the downregulation of ABCB1 at the molecular level has been shown to be less eﬃcacious than blocking the function of ABCB1 (Mohana et al., 2016; Chang et al., 2019). It has been postulated that inhibition of the ABCB1 function may be a potential therapeutic strategy to overcome MDR (Gu et al., 2018). Despite decades of considerable efforts, numerous ABCB1 inhibitors have been shown to signiﬁcantly increase the eﬃcacy of certain anticancer drugs in MDR tumor cells *in vitro* and *in vivo*, but no ABCB1 inhibitors have been approved for clinical use due to the lack of significant clinical efficacy or safety concerns (Bissett et al., 1991; Jackson et al., 2020). Therefore, there is urgency to develop effective, targeted, and nontoxic ABCB1 inhibitors to reverse MDR in cancer.

Antimicrobial peptides (AMPs) are a kind of small molecule peptides with biological activity induced *in vivo* and are important components of the innate immune system (Bala and Kumar, 2014). Over the past decade, more than 3,000 kinds of AMPs have been identified from different species such as bacteria, insects, amphibians, fungi, mammals, higher plants and even humans (Wang, 2020). AMPs generally possess 6–50 amino acid residues, most of which are cationic peptides with net positive charges (Scott and Hancock, 2000; Bradshaw, 2003). Although the mechanism of different peptides may be different under different conditions, it is generally believed that these cationic peptides can selectively interact with the anionic bacterial membrane (Hancock and Rozek, 2002; Marcotte et al., 2003). This suggests that AMPs can also target anionic tumor cell membranes. In fact, with the development of research, some AMPs exhibited an anticancer effect on cancer cells and MDR cancer cells with less toxicity to normal cells (Mai et al., 2001; Hoskin and Ramamoorthy, 2008). At present, there is great interest in AMPs because these so-called natural antibiotics have become therapeutic candidates for new anticancer drugs with different but unclear mechanisms (Shadidi and Sioud, 2003; Wu et al., 2014). Temporin-Pta (FFGSVLKLIPKIL) is an antibacterial peptide from the skin secretions of the Malaysian fire frog *Hylarana picturata* (Conlon et al., 2008). The activity of the natural form of Temporin-Pta was not evaluated due to a low amount of the peptide. But both the L-type and D-type of the synthetic peptide show similar activity in inhibiting the viability of *E. coli* and *S. aureus* USA300 (MRSA) (Mishra and Wang, 2012). Furthermore, the peptide mutant of Pemporin-Pta with S4K, P10R, and L13F mutations was also found to have anti-HIV activities (Wang et al., 2010). In previous studies, we designed a number of AMPs based on the template of Temporin-Pta. The hydrophobicity, amphiphilicity and helicity of AMPs were modified by replacing one or more amino acid residues with other amino acid residues. This project engineered three novel AMPs: HX-12A (FFRKVLKLIRKI), HX-12B (FFRKVLKLIRKIF), and HX-12C (FFRKVLKLIRKIWR) with the replacement of certain antimicrobial peptide amino acids from the template Temporin-Pta. The results of serial biological assays indicated that these peptides have notably improved antimicrobial activities compared to the template Temporin-Pta (Luo et al., 2017).

Discovering new functions of active molecules is an important strategy in drug development. In previous research, we found that the possible site of action of peptide HX-12C was on the negatively charged cell membrane according to the killing kinetics and transmission electron microscopy, making it more difﬁcult for bacteria to develop resistance (Luo et al., 2017; Luo et al., 2018). Therefore, we speculate that these antimicrobial peptides may have antitumor activity because of the negatively charged phospholipids on the surface of tumor cells. And in screening novel peptides that have potential anticancer effects, we found that peptides HX-12A、HX-12B and HX-12C have anticancer activity not only on drug sensitive cancer cells, but also on MDR cancer cells, especially peptide HX-12C. In this study, we first report here peptide HX-12C can reverse the multidrug resistance of tumor cells. The further results showed that the peptide HX-12C had a significant inhibitory effect on ABCB1-mediated drug efflux, resulting in an increase in the concentration of chemotherapeutic drugs in cells and ensuing drug sensitivity. These findings suggested that peptide HX-12C has potential significant ABCB1-overexpressing MDR reversing activity which would allow further design or modification of these peptides.

## Materials and Methods

### Chemicals and Reagents

The antimicrobial peptides were synthesized at and provided by Hunan University of Science and Engineering, Yongzhou, Hunan, China. Bovine calf serum (BS), fetal bovine serum (FBS), Dulbecco’s modified Eagle’s medium (DMEM), trypsin 0.25% and penicillin/streptomycin were purchased from Hyclone (GE Healthcare Life Sciences, Pittsburgh, PA). [^3^H]-paclitaxel (15 Ci/mmol) was purchased from Moravek Biochemicals (Brea, CA). Phosphate buffered saline (PBS), GAPDH Mouse Anti-Human Clone: GA1R, 4′,6-diamidino-2-phenylindole (DAPI), and Alexa Fluor 488 conjugated rabbit anti-mouse IgG secondary antibody were purchased from Thermo Fisher Scientific Inc. (Rockford, IL). Anti-mouse IgG HRP-linked secondary-antibody was purchased from Cell Signaling Technology (Danvers, MA). were obtained from Sigma Chemical Co. (St. Louis, MO). Liquid scintillation cocktail was a product of MP Biomedicals, Inc (St. Ana, CA). The monoclonal anti-P-glycoprotein (MDR) antibody Clone F4 produced in mice, paclitaxel, verapamil, cisplatin, 3-(4, 5-dimethylthiazol-yl)-2, 5-diphenyltetrazolium bromide (MTT), Triton X-100, dimethyl sulfoxide (DMSO), and all other chemicals were purchased from Sigma Chemical Co (St. Louis, MO).

### Cell Lines and Cell Culture

The drug-resistant cell line KB-C2 with high expression of ABCB1 was established by stepwise screening of the parental human epidermoid carcinoma cell line KB-3-1 in increasing concentrations of colchicine and cultured in the medium with 2 mg/mL of colchicine (Akiyama et al., 1985). We had used the paired cell lines for P-gp reversal study since 1993 as KB-C2 drug selected MDR subline is a good model for P-gp associated study. Therefore, we used KB-C2 and its parental cell line KB-3-1 in this study. HEK293/pcDNA3.1 and HEK293/ABCB1 cells lines were established by selection with 2 mg/mL G418 (Enzo Life Sciences, Farmingdale, NY) after transfecting HEK293 with the empty pcDNA3.1 vector or the vector containing full length ABCB1 DNA, respectively (Zhang et al., 2015). All the cell lines were cultured in DMEM containing 1% penicillin/streptomycin and 10% FBS at 37°C with 5% CO_2_. All drug-resistant cell lines were cultured in drug-free medium for more than 2 weeks prior to their use.

### Peptide Synthesis

All peptides used in this study were synthesized by solid phase methods using Fmoc N-terminal protected amino acids, and they were puriﬁed to >95% by reverse-phase high performance liquid chromatography (RP-HPLC). The puriﬁed peptide was conﬁrmed by mass spectrometry analysis.

### Secondary Structure Analysis

The secondary structures of HX-12C was analyzed by Heliquest (http://heliquest.ipmc.cnrs.fr) and Circular Dichroism (CD) assay. The CD assay was performed at room temperature in nitrogen-washed cells using a 2-mm path with the Jasco J-1500 spectropolarimeter. Peptide HX-12C was measured at a concentration of 0.2 mg/mL in PB buffer, 30% TFE/H_2_O or 70% TFE/H_2_O. The configuration of peptide was obtained by analyzing the data with CD deconvolution software.

### Cell Cytotoxicity Determined by MTT Assay

The cytotoxicity of peptides in different cell lines and those of anticancer drugs with or without peptides as mediation agents were determined by modified MTT assay (Fan et al., 2018). Briefly, 5×10^3^ cells per well were evenly seeded in 96-well microplates overnight. For the cytotoxicity assay, peptides and positive control of known substrate anticancer drugs were added in a designated concentration gradient. For the reversal assay, peptides and positive control agents were added 2 h before the addition of anticancer drugs within the specified concentration gradient. The plates were incubated in a culture condition for 68 h followed by the addition of 20 μL MTT solution (4 mg/mL) and further incubation of 4 h. After aspirating the culture medium and MTT solution, the formazan crystals were dissolved with 100 μL DMSO. The absorbance was determined at 570 nm with the accuSkan GO UV/Vis Microplate Spectrophotometer (Fisher Sci., Fair Lawn, NJ).

### Western Blotting Analysis

After treatment with 0, 1, 3, and 6 μM of peptide HX-12C for 72 h in KB-C2 and non-treated KB-3-1, cells were incubated on ice with lysate buffer for 20 min. The protein supernatant was collected after centrifuged for 20 min at 4°C with 12,000g. The concentration of protein was determined by the method of protein assay based on Bicinchoninic Acid (BCA) (Thermo Scientific, Rockford, IL). Equal amount of each protein sample was loaded and separated by SDS-polyacrylamide gel electrophoresis. After that, the gel was transferred to the polyvinylidene difluoride (PVDF) membrane followed by blocking with 5% dry milk for 2 h. Then, the membrane was incubated overnight at 4°C with primary antibodies (1:1000 dilution for both anti-P-glycoprotein and anti-GAPDH antibodies), and then incubated with the secondary HRP-labeled antibody. The signal was detected by enhanced chemiluminescence, and the protein expression was quantified by ImageJ software (NIH, Bethesda, MD, USA).

### Immunofluorescence Assay

KB-C2 and KB-3-1 cells were seeded in 24-well plates at 1×10^4^ cells per well and cultured overnight. To test the effects of peptide samples, the cells were cultured with 3 μM peptide HX-12C for 0, 24, 48, and 72 h, and then the cells were fixed in 4% formaldehyde for 15 min and permeabilized with 0.25% Triton X-100 for 15 min. After incubated with BSA (6% with PBS) for 1 h, the cells continued to be incubated with monoclonal anti-P-glycoprotein Clone F4 primary antibody with dilution of 1:1000 overnight at 4°C, followed by further incubation with Alexa Fluor 488 secondary antibody in dark for 2 h. DAPI solution was used to counterstain the nuclei. Nikon TE-2000S fluorescence microscope (Nikon Instruments Inc., Melville, NY, USA) was used to collect immunofluorescent images.

### Accumulation and Efflux Assay

Drug accumulation and efflux assays were performed as previously described (Fan et al., 2018). For the drug accumulation assay, KB-C2 and KB-3-1 cells were seeded at 1×10^4^ cells per well into 24-well plates and incubated at 37°C overnight. After cells were incubated in the presence or absence of peptide HX-12C and positive reversal agent for 72 h, the medium was replaced by medium containing 5 μM [^3^H]-paclitaxel with peptide HX-12C or positive reversal agent. The drug medium was discarded after 2 h incubation, then cells were washed three times with ice-cold PBS and transferred to the scintillation fluid. The drug efflux assay was performed after discarding the drug medium, then cells were washed three times with ice-cold PBS and treated with the medium containing peptide HX-12C or positive reversal agent. There were three different time points (30, 60, and 120 min) at which after changing the medium, the cells were washed three times with ice-cold PBS and collected and transferred to the scintillation fluid. The Packard TRI-CARB1 190`A liquid scintillation analyzer was used to measure radioactivity. A parallel well of cells for each group was set up for cell number counting at the end of assay for the purpose of data normalization.

### ABCB1 ATPase Assay

The ABCB1 ATPase activity was measured based on vanadate-sensitive principle as previously described (Zhang et al., 2016). The High Five insect cells membrane vesicles (BD Biosciences, San Jose, CA, USA) were first incubated for 5 min in ATPase assay buffer at 37°C with or without 0.3 mM vanadate. Then peptide HX-12C at a concentration of 0 to 40 μM was adding to the assay buffer and incubated at 37°C for 3 min. After adding 5 mM Mg-ATP with a total volume of 0.1 mL, the ATPase reaction was initiated until stopped by adding 100 μL 5% SDS solution after incubation at 37°C for 20 min. The amount of inorganic phosphate (IP) was used to calculate the ATPase activity of ABCB1, detected at 800 nm using a spectrophotometer.

### Molecular Docking Analysis

A molecular docking study was performed by Sybyl/Surflex-dock based on crystal structures of ABCB1 (Qin et al., 2017; Qin et al., 2018). Peptide HX-12C was depicted by Sybyl/Sketch module (Tripos Inc.), optimized by applying Powell’s method with Tripos force field with convergence criterion set at 0.05 kcal/(Å mol), and assigned by the Gasteiger-Hückel method. The crystal structure of ABCB1 was obtained from RCSB Protein Data Bank (PDB ID: 4M2T). Using ligand-based mode, peptide HX-12C was docked into the active sites of ABCB1. The ligand was removed, and hydrogen was added and minimized using Tripos force field and Pullman charges. Other docking parameters remained default values.

### Statistical Analysis

All experiments were performed independently at least three times, and the results were expressed as mean ± Standard Deviation (SD). The statistical difference between the two groups was determined by one-way ANOVA and P values less than 0.05 were considered significant. The *post hoc* test was determined by Dunnett’s test.

## Results

### Prediction and Analysis of Secondary Structure of Peptide HX-12C

**Figure 1:**
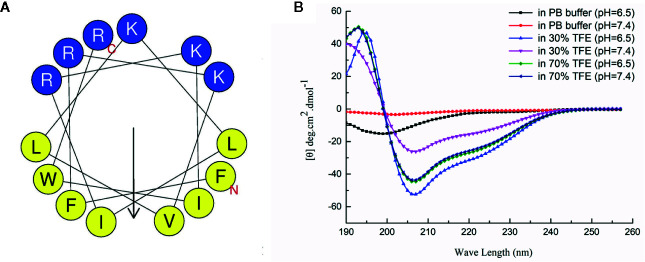
Secondary structure analysis of HX-12C. **(A)** The secondary structure of HX-12C was a typical amphiphilic structure. The hydrophobic amino acid residues and hydrophilic residues are distributed on both sides of the helix. **(B)** The circular dichroism analysis structure of HX-12C. In the PB buffer, the coil is irregular, while in the presence of a cosolvent, it forms a typical spiral structure (Luo et al., 2017).

The secondary structure was predicted by the secondary structure prediction service (HeliQuest), and the secondary structure of the antibacterial peptide was analyzed by circular dichroism. As shown in **Figure 1**, the predicted secondary structure of HX-12C has very typical amphiphilic molecular characteristics, and the hydrophobic and hydrophilic surfaces are regularly distributed on both sides of the polypeptide helix. The analysis results of circular dichroism confirmed the prediction. The structure of circular dichroism was an irregular secondary structure in the water phase (0.01mol/L PB buffer), and a typical α-helix structure is formed in the membrane phase (30% TFE or 70% TEF). Moreover, the acidic environment was more conducive to the formation of the helix structure. It is suggested that the acidic microenvironment of tumor tissue will be more conducive to the interaction between antimicrobial peptides and tumor cell membrane.

### Effects of Peptides on ABCB1-Overexpressing Cell Lines

**Figure 2:**
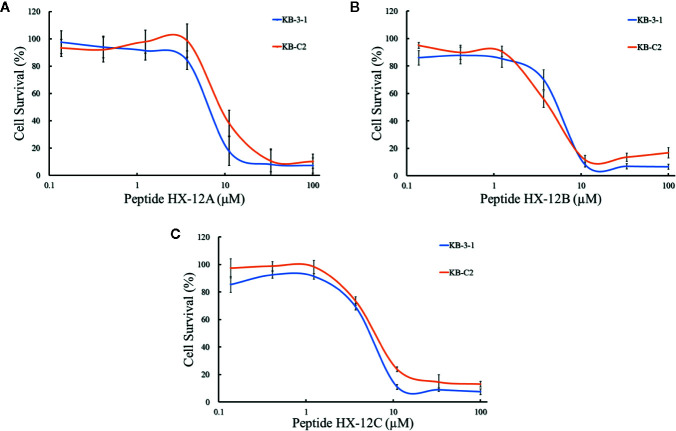
The concentration-survival curves of three peptides HX-12A **(A)**, HX-12B **(B)**, and HX-12C **(C)** on drug-selected ABCB1-overexpressing cell lines (KB-C2) and their parental cell lines (KB-3-1) with gradient concentrations of peptide samples. Data points with error bars represent the mean ± SD from three independently repeated experiments.

To investigate the effects of the three peptides (HX-12A, HX-12B, and HX-12C) on ABCB1 transporter, we first tested the sensitivity of ABCB1-overexpressing cancer cells (KB-C2) to these three peptides. It was worth noting that the results of the MTT assay showed that the IC_50_ values of peptides HX-12A, HX-12B, and HX-12C in ABCB1-overexpressing cancer cells (KB-C2) were 6.45 μM, 7.61μM, and 6.06 μM, respectively. The IC_50_ values of the peptides in parental cells (KB-3-1) were 9.18 μM, 5.42 μM, and 7.53μM, respectively (**Figure 2**). More significantly, the results of the MTT assay showed that these three peptides would not produce significant cytotoxicity (at least 85% cell survival) at a concentration lower than 3 μM.

### Peptides Sensitize ABCB1-Overexpressing Cells to Chemotherapeutic Drugs

To determine whether the peptides (HX-12A, HX-12B, and HX-12C) can reverse multi-drug resistance mediated by ABCB1 transporter, we tested the sensitivity of drug-induced resistant tumor cell line (KB-C2), the transfected ABCB1-overexpressing cell lines (HEK293/ABCB1) and their parental cell lines (KB-3-1 and HEK293/pcDNA3.1) to the peptides. The results are shown in **Tables 1** and **2**, where the IC_50_ values of ABCB1 overexpressing cells (KB-C2 and HEK293/ABCB1) to ABCB1 substrates, paclitaxel and doxorubicin, were significantly higher than those of their parental cells (KB-3-1 and HEK293/pcDNA3.1). In addition, compared with the corresponding parental cells (KB-3-1 and HEK293/pc DNA3.1), there was no significant change in IC_50_ values of cisplatin (a non-substrate of ABCB1) in KB-C2 or HEK293/ABCB1 cells. These results indicate that peptide HX-12C can reverse the MDR of cancer cells mediated by ABCB1 overexpression.

**Table 1:** Reversal effect of three peptides on drug-selected ABCB1-overexpressing cells.

Treatment	IC_50_^1^(μM) (RF^2^)	
	KB-3-1	KB-C2
Paclitaxel	0.002 ± 0.0007 (1.00)	1.430 ± 0.241 (715.00)
+ HX-12A (3 μM)	0.005 ± 0.0004 (2.50)	1.389 ± 0.356 (694.50)
+ HX-12B (3 μM)	0.001 ± 0.0006 (0.50)	0.542 ± 0.097 (271.00)
+ HX-12C (3 μM)	0.003 ± 0.0007 (1.50)	0.031 ± 0.002^***^ (15.50)
+ Verapamil (3 μM)	0.001 ± 0.0002 (0.50)	0.030 ± 0.004^***^ (15.00)
Doxorubicin	1.186 ± 0.279 (1.00)	69.79 ± 4.637 (58.84)
+ HX-12A (3 μM)	1.099 ± 0.169 (0.93)	65.41 ± 0.281 (55.15)
+ HX-12B (3 μM)	1.042 ± 0.211 (0.88)	40.21 ± 0.206 (33.90)
+ HX-12C (3 μM)	1.012 ± 0.060 (0.85)	1.454 ± 0.015^***^ (1.23)
+ Verapamil (3 μM)	0.897 ± 0.143 (0.76)	0.734 ± 0.165^***^ (0.62)
Cisplatin	1.793 ± 0.476 (1.00)	2.025 ± 0.428 (1.13)
+ HX-12A (3 μM)	1.687 ± 0.398 (0.94)	2.503 ± 0.336 (1.40)
+ HX-12B (3 μM)	1.593 ± 0.512 (0.89)	2.096 ± 0.389 (1.17)
+ HX-12C (3 μM)	1.693 ± 0.452 (0.94)	2.087 ± 0.233 (1.16)
+ Verapamil (3 μM)	1.676 ± 0.478 (0.93)	2.107 ± 0.361 (1.18)

^1^IC_50_ values are represented as mean ± SD of at least three independent experiments performed in triplicate. ^2^RF, resistant fold, calculated by the IC_50_ values in the drug-induced ABCB1-overexpressing cancer cell line KB-C2 divided by the IC_50_ values in the parental cancer cell line KB-3-1. ^***^represents p < 0.001, compared to the value of KB-C2 control group.

**Table 2:** Reversal effect of three peptides on transfected ABCB1-overexpressing cells.

Treatment	IC_50_^1^ (μM) (RF^2^)	
	HEK293/pcDNA3.1	HEK293/ABCB1
Paclitaxel	1.449 ± 0.121 (1.00)	30.924 ± 4.665 (21.34)
+ HX-12A (3 μM)	1.405 ± 0.285 (0.97)	22.117 ± 1.199 (15.26)
+ HX-12B (3 μM)	1.302 ± 0.255 (0.90)	20.190 ± 1.047 (13.93)
+ HX-12C (3 μM)	1.446 ± 0.462 (1.00)	1.501 ± 0.342^***^ (1.04)
+ Verapamil (3 μM)	1.486 ± 0.377 (1.03)	1.708 ± 0.147^***^ (1.18)
Doxorubicin	1.235 ± 0.571 (1.01)	31.233 ± 5.406 (25.29)
+ HX-12A (3 μM)	1.206 ± 0.405 (0.98)	25.327 ± 4.318 (20.51)
+ HX-12B (3 μM)	1.221 ± 0.488 (0.99)	22.374 ± 3.774 (18.12)
+ HX-12C (3 μM)	1.420 ± 0.453 (1.09)	1.182 ± 0.253^***^ (0.94)
+ Verapamil (3 μM)	1.271 ± 0.358 (1.03)	1.245 ± 0.265^***^ (1.00)
Cisplatin	2.214 ± 0.541 (1.00)	2.665 ± 0.208 (1.20)
+ HX-12A (3 μM)	2.337 ± 0.438 (1.06)	2.525 ± 0.284 (1.14)
+ HX-12B (3 μM)	2.403 ± 0.389 (1.09)	2.692 ± 0.176 (1.22)
+ HX-12C (3 μM)	2.464 ± 0.144 (1.11)	2.184 ± 0.317 (0.99)
+ Verapamil (3 μM)	2.128 ± 0.238 (0.96)	2.287 ± 0.138 (1.03)

^1^IC_50_ values are represented as mean ± SD of at least three independent experiments performed in triplicate. ^2^RF, resistant fold, calculated by the IC_50_ values in the transfected ABCB1-overexpressing cell line HEK293/ABCB1 divided by the IC_50_ values in the empty-vector transfected cell line HEK293/pcDNA3.1. ^***^represents p < 0.001, compared to the value of HEK293/ABCB1 control group.

### Effects of Peptide HX-12C on the Expression Level and Cellular Localization of ABCB1

**Figure 3:**
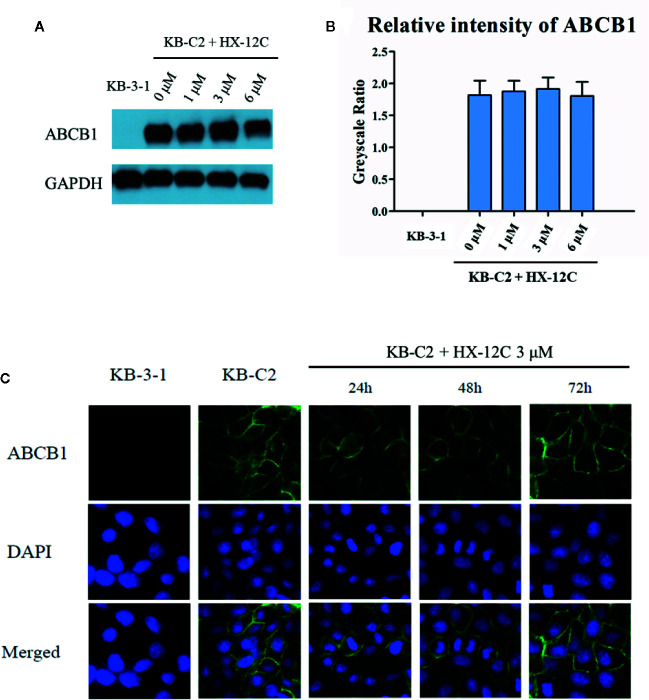
The effect of peptide HX-12C on the expression level and subcellular localization of ABCB1 protein in KB-3-1 and KB-C2 cells. **(A)** The expression of ABCB1 protein after incubation with different concentrations of peptide HX-12C for 72 h in KB-C2 cells. KB-3-1 cells without any treatment used as the negative control of ABCB1 protein expression. GAPDH was used as loading control. **(B)** The relative intensity of the expression of ABCB1 to the expression of GAPDH. Expression level quantification by gray scale values calculated by ImageJ software. **(C)** The expression of ABCB1 protein after incubation with 3 μM peptide HX-12C for 0, 24, 48, and 72 h. DAPI was used to counterstain the nuclei to locate the cells. Pictures have been modified by Photoshop software for merged comparison.

To investigate the possible mechanism of the sensitivity of ABCB1-overexpressed cells to anticancer drugs caused by peptide HX-12C, we studied the effect of peptide HX-12C on the expression level and cellular localization of the ABCB1 protein in ABCB1-overexpressing cells (KB-C2) and their parental cells (KB-3-1). Western blot analysis was used to confirm whether peptide HX-12C had influence on the expression of ABCB1 transporter. As shown in **Figures 3A, B**, different concentrations (0, 1, 3, 6 μM) of peptide HX-12C treatment had no significant effect on the expression of ABCB1 protein in KB-C2 cells. Immunofluorescence staining was carried out after treatment of cells with different incubation times of peptide HX-12C. As shown in **Figure 3C**, compared to parental cells KB-3-1, ABCB1 protein was significantly expressed on the cell membrane of KB-C2. The fluorescent intensity of ABCB1 protein in KB-C2 also remained unchanged after the treatment of peptide HX-12C, which was consistent with Western blot results. Furthermore, the peptide HX-12C had no significant effect on the subcellular distribution pattern of ABCB1 protein on KB-C2 cell membrane. These results indicated that the reversal of MDR by peptide HX-12C was not caused by decreased protein expression or change in protein location.

### Peptide HX-12C Affects [^3^H]-Paclitaxel Accumulation and Efflux

**Figure 4:**
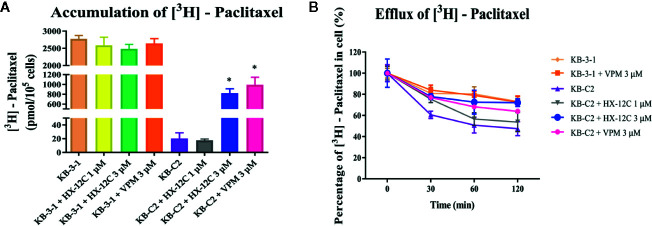
The accumulation and efflux activity of peptide HX-12C in ABCB1-overexpressing KB-C2 cells and their parental KB-3-1 cells. **(A)** Effects of peptide HX-12C on intracellular accumulation of [^3^H]-paclitaxel in ABCB1-overexpressing KB-C2 cells and their parental KB-3-1 cells. Columns with error bar represent mean ± SD. ^*^, represents p < 0.05, compared to the KB-C2 control group. VPM, verapamil. **(B)** Effects of peptide HX-12C on efflux of [^3^H]-paclitaxel in ABCB1-overexpressing KB-C2 cells and their parental KB-3-1 cells. VPM, verapamil. The points with error bars represent the mean ± SD from three independently repeated experiments.

Previous data have confirmed that peptide HX-12C did not significantly affect the expression level and cell location of ABCB1 protein, so we speculated that peptide HX-12C may reverse drug resistance by inhibiting the ABCB1 protein’s efflux function. Therefore, we tested the effect of peptide HX-12C on the accumulation and efflux of chemotherapeutic drugs in ABCB1-overexpressing KB-C2 cells and their parental KB-3-1 cells. Intracellular [^3^H]-paclitaxel was measured in ABCB1-overexpressing cell line (KB-C2) and their parental cell line (KB-3-1) in the presence or absence of peptide HX-12C. The result is shown in **Figure 4A**. The level of [^3^H]-paclitaxel in ABCB1-overexpressing cells (KB-C2) was approximately 100-fold lower than that of their parental cells (KB-3-1) after 2 hours of incubation. Compared with the control group, the intracellular concentration of [^3^H]-paclitaxel in KB-C2 cells was significantly increased with peptide HX-12C (3 μM) treatment. The effect of peptide HX-12C (3 μM) on the accumulation of [^3^H]-paclitaxel is comparable to that of verapamil (3 μM), which is an inhibitor of ABCB1. In addition, we evaluated the effect of peptide HX-12C on the efflux of [^3^H]-paclitaxel in ABCB1-overexpressing cells (KB-C2), and the results are shown in **Figure 4B**. There was no significant change in intracellular [^3^H]-paclitaxel level in KB-3-1 cells after treated for 2 hours with an inhibitor, nor did the inhibitors alter the efflux function in KB-3-1 cells. However, the level of intracellular [^3^H]-paclitaxel in KB-C2 cells decreased by about 50% without inhibitor treatment. This demonstrates that the efflux function of KB-C2 cells was effectively inhibited by treatment with peptide HX-12C (3 μM).

### Peptide HX-12C Stimulates ATPase Activity

**Figure 5:**
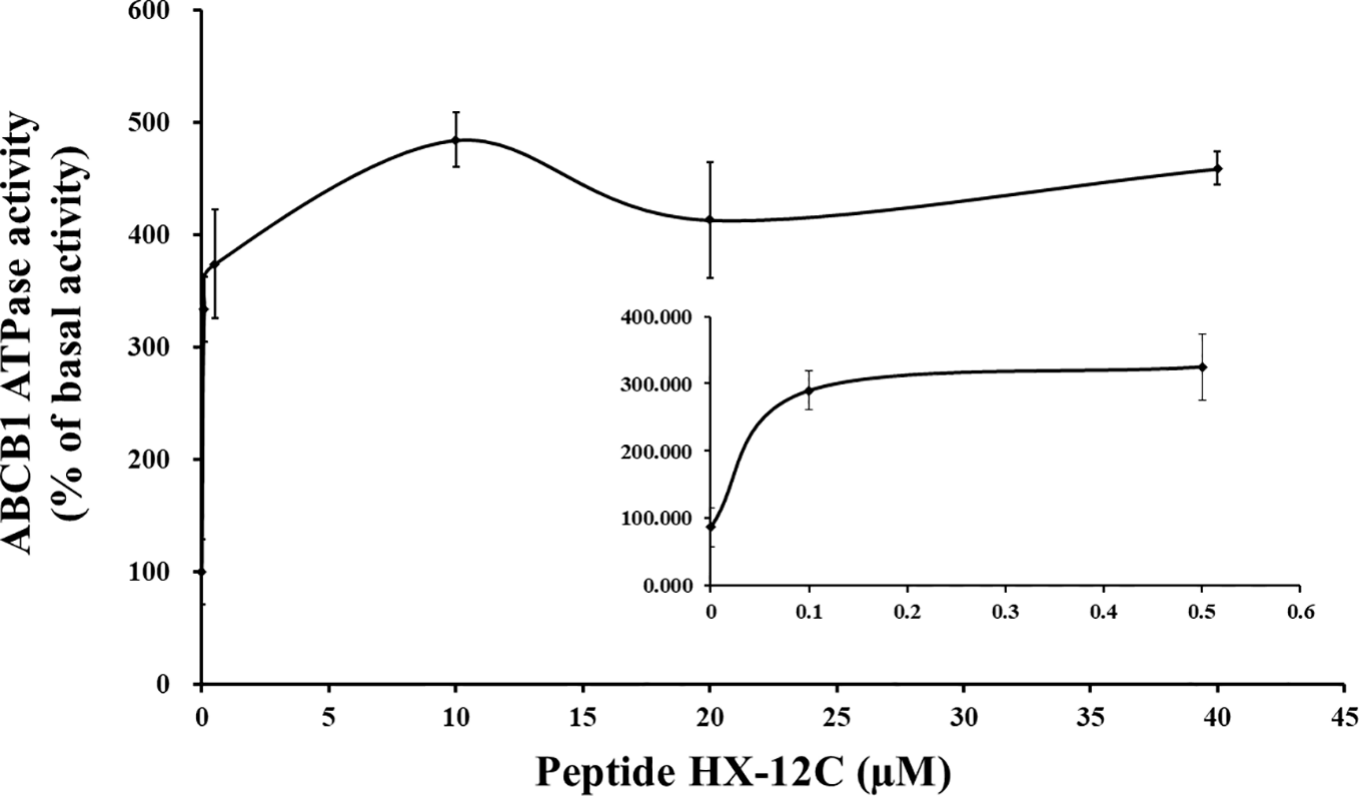
Effect of peptide HX-12C on the ATPase activity of ABCB1. The sensitive ATPase activity of ABCB1 in insect cell membrane vesicles was determined in the presence of a series of different concentrations of peptide HX-12C. The small inner figure plotted the lower concentrations (0–0.5 μM) of peptide HX-12C versus ATPase activity. The points with error bars represent the mean ± SD from three independently repeated experiments.

Generally, most ABCB1 transporter substrates and inhibitors activate the ATPase activity of ABCB1, while only a few compounds inhibit it (Ambudkar et al., 1999; Chufan et al., 2016). To evaluate the effect of peptide HX-12C on the ABCB1 ATPase activity, five insect cell membrane vesicles overexpressing ABCB1 were used in the presence of a series of different concentrations of peptide HX-12C, under the condition of inhibiting the activity of other major membrane ATPases. As shown in **Figure 5**, the results indicated that peptide HX-12C can stimulate the ATPase activity of ABCB1 in a concentration-dependent manner, and the maximal stimulation was 4.7-fold of the basic activity. The concentration of peptide HX-12C required for 50% stimulation of ATPase activity of ABCB1 was 0.65 μM, which was much lower than the reversal concentration in the cytotoxicity assay. These results indicated that peptide HX-12C may interact with drug substrate binding sites and affect the ATPase activity of ABCB1.

### Analysis of Peptide HX-12C-ABCB1 Binding by a Molecular Docking Study

**Figure 6:**
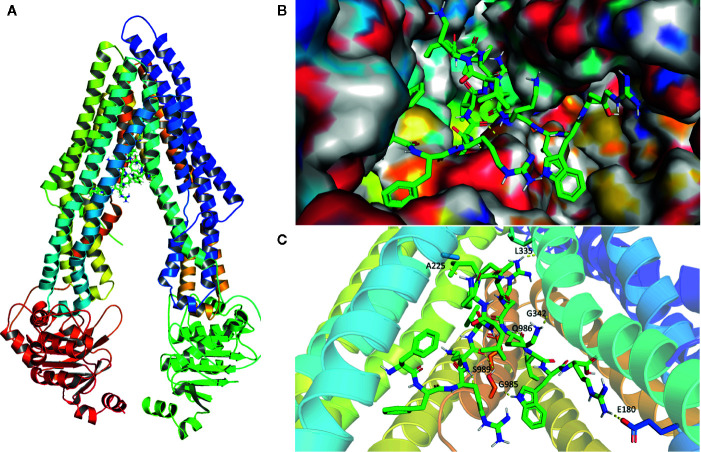
Model structure showing the binding mode of peptide HX-12C to ABCB1. **(A)** Domains and positions of ABCB1 binding with peptide HX-12C. **(B)** Structures showing the surface of peptide HX-12C-ABCB1 complex. Peptide HX-12C is rendered in green. **(C)** The predicted binding mode of peptide HX-12C with ABCB1. The key residues interacting with peptide HX-12C are rendered as sticks. The hydrogen bonds are shown as yellow dotted lines.

To further explore the potential binding modes and rationalize the observed efficacy of peptide HX-12C, a molecular docking study was performed. As shown in **Figure 6**, peptide HX-12C tightly bonded to active sites of ABCB1. This peptide formed eight hydrogen bonds with residues E180, A225, L335, G342, G985, Q986, and S989 in ABCB1, which may play an important role in improving the binding affinity of peptide HX-12C to ABCB1. It is worth pointing out that the salt bridge involving arginine in this peptide and E180 in ABCB1 may contribute to high binding affinity. Peptide HX-12C is buried in hydrophobic contacts with other residues such as F299, F990, L232, and I302 in ABCB1.

## Discussion

Despite extensive efforts, MDR is still a major obstacle to success of chemotherapy in cancer treatment. Given the dominant role of ABC transporters, the development of selective, potent and safe reversal agents, which could inhibit the efflux functions of ABC transporters, is urgently needed for the progress of advanced chemotherapy. ABCB1 is a typical transporter in the ABC protein superfamily and is expressed broadly in organs and tissues in the human body. Therefore, the combination of ABCB1 inhibitors and chemotherapy drugs is considered a promising strategy to overcome ABCB1-mediated multidrug resistance. To date, ABCB1 inhibitors have developed from the first generation to the fourth generation (Zhang et al., 2014; Karthikeyan and Hoti, 2015; Zhang et al., 2016). However, there are some challenges in developing ABCB1 inhibitors, such as the ubiquitous expression of ABCB1 (present in both normal and tumor tissues), complicated drug-drug interactions, and neurotoxicity. The change in direction of discovering effective reversal agents from traditional tyrosine kinase inhibitors to other kinds of compounds may be an innovative way to address this problem. Antimicrobial peptides are novel anticancer drugs with less possibility of causing cancer cell drug resistance. Some primary differences between the cell membranes of neoplastic and normal cells are the reasons for the elective higher cytotoxicity of antimicrobial peptides in cancer cells than in healthy cells. Therefore, more and more antimicrobial peptides have been used in cancer treatment because of their unique therapeutic effects, especially cationic peptides (Leuschner and Hansel, 2004; Wu et al., 2009; Hsiao et al., 2013). This present study investigated the anticancer and the possible MDR reversal effects of some specially designed antimicrobial peptides.

In this study, we detected the secondary structure of the peptides. The circular dichroism spectra indicated that peptide HX-12C randomly curls in aqueous solution, but adopts a helical conformation in a membrane-like environment, especially in an acidic environment, which suggested that the polypeptide may have anticancer activity, as confirmed by cytotoxicity assays. Our data showed that these peptides could kill the resistant cell lines at a similar concentration level as compared with their IC_50_ levels against the parental cell lines. However, only peptide HX-12C potently sensitized ABCB1-overexpressing cells, induced both by drugs and gene-transfection, to ABCB1 substrates paclitaxel and doxorubicin. Furthermore, peptide HX-12C had no effect on the sensitivity of the cells tested with cisplatin. These results suggest that the reversal effect of peptide HX-12C could be specific for ABCB1.

The most common ways that the reversal agents re-sensitize drug-resistant cells include blocking the function of transporters and down-regulating the expression of transporters (Kim et al., 2014; Sui et al., 2014; Kathawala et al., 2015). The Western blotting and immunoblotting analyses showed that peptide HX-12C did not affect the expression of ABCB1 protein in overexpressing cells. Moreover, the results of immunofluorescence assay supported the postulate that peptide HX-12C at 3 μM did not alter the cellular localization of ABCB1 transporter in MDR cells at 72 h. These results suggested that this peptide may block the function of the ABCB1 transporter. The mechanism of ABC transporter-induced MDR is due to overexpression of ABC transporter enhancing the efflux of anticancer drugs, therefore reducing the intracellular concentration of anticancer drugs. We examined the effect of peptide HX-12C on the accumulation and efflux of [^3^H]-paclitaxel in ABCB1-overexpressing cells. The results indicated that peptide HX-12C significantly enhanced the intracellular accumulation of [^3^H]-paclitaxel in ABCB1-overexpressing cells by reducing its efflux at a level which was comparable to that of verapamil. These results indicated that peptide HX-12C reversed ABCB1-mediated drug resistance by inhibiting the function of ABCB1 transporters directly.

The interaction between peptide HX-12C and ABCB1 was investigated by ATPase assay. Generally, the ATPase activity of ABCB1 transporters is stimulated in the presence of transport substrates or competitive inhibitors, and the data showed that peptide HX-12C can stimulate the ATPase activity of ABCB1 in a concentration-dependent manner, and the maximal stimulation was 4.7-fold of the basic activity. These results suggested that peptide HX-12C might be a potential substrate of ABCB1 transporter. Therefore, peptide HX-12C may be a competitive inhibitor of ABCB1 by competing with other drug substrates.

The drug-binding pocket of ABCB1 located at the interface between the two transmembrane domains is formed by hydrophobic and aromatic residues (Li et al., 2016). It is not surprising that lipophilicity is the main contribution to effective ABCB1 inhibitory activity (Raub, 2006). In addition, the structure-activity relationship analysis of ABCB1 inhibitors clearly indicated the importance of multiple factors including hydrophobic, positive ionizable groups, aromatic ring center, and hydrogen bond acceptor for ABCB1 inhibitors (Ekins, 2010). Interestingly, peptide HX-12C exhibits all of these pharmacophoric features, which explains its affinity to the central hydrophobic cavity of ABCB1. Our molecular docking studies have shown the possible binding modes between peptide HX-12C and ABCB1. Although the binding data for peptide HX-12C within ABCB1 active sites is yet to be experimentally validated, the binding model of peptide HX-12C within ABCB1 active sites may provide guidance for the development of ABCB1 inhibitors.

In conclusion, this study first showed that peptide HX-12C inhibited ABCB1-mediated drug efflux, resulting in an increase in the concentration of antitumor drugs and drug sensitivity. Further research showed that peptide HX-12C can stimulate the ATPase activity of ABCB1. An animal study with a xenograft mice model will be needed to determine whether peptide HX-12C can reverse MDR *in vivo*. At a minimum, this study first showed a new function of peptide HX-12C to overcome ABCB1-mediated multidrug resistance by inhibiting the function of ABCB1, and provides a potential clue for the development of ABCB1 inhibitors.

## Conclusions

In conclusion, this study first reports that peptide HX-12C could re-sensitize ABCB1-overexpressing cells by inhibiting the efflux function without affecting the expression level or localization of ABCB1. This study provides a promising therapeutic strategy to overcome ABCB1-mediated drug resistance of tumor cells. However, the clinical therapeutic effect still needs to be explored in further studies by *in vivo* xenograft animal models and clinical trials. Further studies are also warranted to confirm the structure of these peptides and whether they could be contributed to improving clinical outcomes in patients receiving chemotherapy.

## Data Availability Statement

The raw data supporting the conclusions of this article will be made available by the authors, without undue reservation.

## Author Contributions

XL: Conception and design. Q-XT: Acquisition of data. J-YD: Structure prediction and molecular docking analysis. D-HY: Revising the manuscript. MW: Analysis and interpretation of data. WD: Writing the manuscript. J-JQ: Technical support. Z-NL: Making figures. J-QW: Making tables. ZQ: Development of methodology. Z-SC: Administrative and material support.

## Funding

This work was supported by the Natural Science Foundation of Hunan Province (2019JJ30011, 2019JJ50195 and 2018WK2093), funds from National Natural Science Foundation of China (81903842) and Department of Pharmaceutical Sciences, St. John’s University.

## Conflict of Interest

The authors declare that the research was conducted in the absence of any commercial or financial relationships that could be construed as a potential conflict of interest.
